# Serotonin Transporter Deficiency Induces Metabolic Alterations in the Ileal Mucosa

**DOI:** 10.3390/ijms25084459

**Published:** 2024-04-18

**Authors:** Nathan Calzadilla, Dulari Jayawardena, Aisha Qazi, Anchal Sharma, Kai Mongan, Shane Comiskey, Abhijith Eathara, Seema Saksena, Pradeep K. Dudeja, Waddah A. Alrefai, Ravinder K. Gill

**Affiliations:** 1 Department of Biomedical Engineering, University of Illinois Chicago, Chicago, IL 60607, USA; ncalza3@uic.edu; 2 Division of Gastroenterology & Hepatology, University of Illinois Chicago, Chicago, IL 60612, USA; djayaw2@uic.edu (D.J.); aqazi7@uic.edu (A.Q.); anchals@uic.edu (A.S.); kmongan@neomed.edu (K.M.); shanec@uic.edu (S.C.); aeatha2@uic.edu (A.E.); saksena@uic.edu (S.S.); pkdudeja@uic.edu (P.K.D.); walrefai@uic.edu (W.A.A.); 3 Jesse Brown VA Medical Center, Chicago, IL 60612, USA

**Keywords:** metabolomics, lipid metabolism, metabolic syndrome, serotonin, serotonin transporter

## Abstract

Serotonin transporter (SERT) deficiency has been implicated in metabolic syndrome, intestinal inflammation, and microbial dysbiosis. Interestingly, changes in microbiome metabolic capacity and several alterations in host gene expression, including lipid metabolism, were previously observed in SERT^−/−^ mice ileal mucosa. However, the precise host or microbial metabolites altered by SERT deficiency that may contribute to the pleiotropic phenotype of SERT KO mice are not yet understood. This study investigated the hypothesis that SERT deficiency impacts lipid and microbial metabolite abundances in the ileal mucosa, where SERT is highly expressed. Ileal mucosal metabolomics was performed by Metabolon on wild-type (WT) and homozygous SERT knockout (KO) mice. Fluorescent-activated cell sorting (FACS) was utilized to measure immune cell populations in ileal lamina propria to assess immunomodulatory effects caused by SERT deficiency. SERT KO mice exhibited a unique ileal mucosal metabolomic signature, with the most differentially altered metabolites being lipids. Such changes included increased diacylglycerols and decreased monoacylglycerols in the ileal mucosa of SERT KO mice compared to WT mice. Further, the ileal mucosa of SERT KO mice exhibited several changes in microbial-related metabolites known to play roles in intestinal inflammation and insulin resistance. SERT KO mice also had a significant reduction in the abundance of ileal group 3 innate lymphoid cells (ILC3). In conclusion, SERT deficiency induces complex alterations in the ileal mucosal environment, indicating potential links between serotonergic signaling, gut microbiota, mucosal immunity, intestinal inflammation, and metabolic syndrome.

## 1. Introduction

While serotonin (5-hydroxytryptamine; 5-HT) is best known for its role as a neurotransmitter, serotonin has emerged as a key regulator of intestinal functions, including electrolyte secretion, absorption, blood flow, and motility [1,2,3,4]. Indeed, it has been shown that 95% of 5-HT is synthesized and stored in enterochromaffin cells in the gut, which then release 5-HT through mechanosensitive Piezo2 channels [5,6,7,8]. At physiologic pH, 5-HT is a positively charged molecule that cannot cross cell membranes through passive diffusion and thus requires an active transporter for cellular uptake and subsequent degradation by intracellularly localized enzymes such as monoamine oxidases. Therefore, 5-HT availability is primarily controlled by the serotonin transporter (SERT), a 12 transmembrane domain transporter that removes extracellular 5-HT through a Na^+^/Cl^−^ dependent process [9,10]. SERT is expressed in epithelial cells, neurons, immune cells, and platelets [11,12]. As such, SERT has been implicated in several neurological and neuropsychiatric disorders, such as depression, Alzheimer’s, Parkinson’s disease, autism, ADHD, bipolar disorder, Tourette’s syndrome, visceral pain, and anxiety [6,13,14,15]. More specifically, intestinal epithelial cell SERT has been implicated in several disease states related to intestinal inflammation, such as enteric infections, animal models of colitis/ileitis, and inflammatory bowel disease (IBD) [16,17,18,19]. Consequently, SERT-deficient mice have been generated to better understand how alterations in serotonin reuptake influence serotonin (5-HT)-dependent physiology and pathology [20]. SERT knockout (KO) mice exhibit anxiety-like behavior, reduced bone density, and a metabolic phenotype resembling type 2 diabetes mellitus, underscoring the broad implications of disrupted 5-HT regulation [21,22,23,24].

SERT downregulation has been observed in inflammatory bowel diseases [25]. Additionally, the SERT KO model has been shown to have increased susceptibility to 2,4,6-trinitrobenzene sulfonic acid (TNBS) and IL-10 knockout-induced intestinal inflammation, reinforcing the need to better understand the effects of intestinal SERT [26,27]. Previous studies have shown that SERT KO mice exhibit gut microbial dysbiosis and changes in metabolic functions of the intestinal microbiome that strongly correlate with altered lipid metabolism gene pathways in the ileum [28]. However, the precise mechanisms underlying these observations, both in terms of specific gut microbial changes and alterations in host metabolites, remain elusive.

Metabolomic approaches have been utilized to better understand the bidirectional communication between the gut microbiota and the host, especially in the context of intestinal inflammation [29,30,31,32,33,34,35,36,37]. Regarding serotonin, previous work by our group has shown that serum 5-HT levels can differentiate activity states in patients with Crohn’s disease, a subset of IBD [38]. Our group has previously performed metagenomics shotgun sequencing and taxonomic and functional profile assessment on SERT KO mice, which has shown that there were several lipid-related genes that were differentially expressed in the ileal mucosa of SERT KO mice compared to WT mice [28,39]. Notably, acyl-CoA thioesterase-1, 3-Hydroxy-3-methylglutaryl-Coenzyme a synthase-2, 3-hydroxybutyrate dehydrogenase (type 2), and palmitoyl-protein thioesterase-1 were also more than two-fold up-regulated in SERT KO ileal mucosa. However, the effects of such changes manifested as metabolic alterations caused by SERT deficiency at the level of the intestinal mucosa are currently unknown. The current study, thus, performed ileal mucosal metabolomic profiling in SERT-deficient mice, given that intestinal SERT is expressed at the highest levels in the small intestine [12]. The objectives of this study were to identify differentially altered host and microbial metabolites in SERT KO mice ileal mucosa to better understand the consequences of SERT deficiency in disorders associated with altered 5-HT availability, to investigate how SERT deficiency affects intestinal immune populations, and to establish a foundation for future studies investigating the interplay between serotonin signaling, intestinal metabolism, and immune function. SERT-deficient mice exhibit transcriptomic alterations and gut microbial dysbiosis with changes in lipid-related and aryl hydrocarbon receptor (AhR) pathways [28,39]. Thus, this study was driven by the hypothesis that ileal mucosa in SERT-deficient mice exhibits distinct alterations in the composition of lipids and metabolome, affecting immune cell populations within the ileum. Such changes are anticipated to shed light on the underlying mechanisms linking serotonin transporter deficiency to metabolic syndrome, as well as the increased susceptibility to intestinal inflammation observed in SERT-deficient mice.

## 2. Results

### 2.1. SERT Deficiency Leads to Distinct Metabolomic Profile in the Ileal Mucosa Characterized by Alterations in Lipid Metabolism

**Figure 1:**
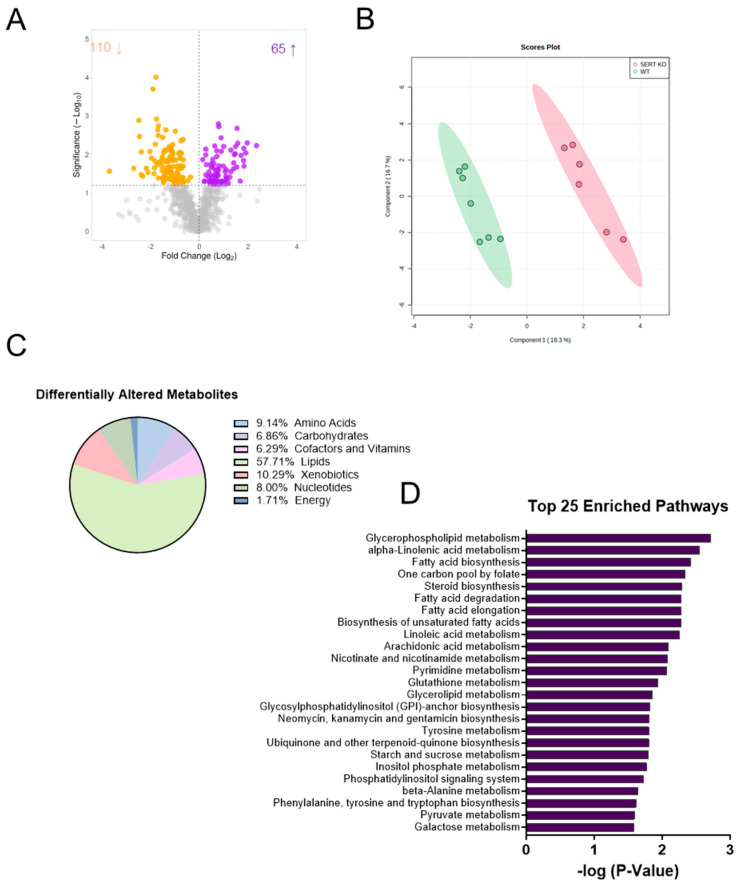
SERT KO mice exhibit a unique ileal mucosal metabolomic profile. (**A**) Volcano plot (log_2_ fold change of metabolites in SERT knockout (KO) compared to wild-type (WT) mice was plotted against the −log_10_ of the *p*-value) where the up-facing arrow indicates the number of significantly increased metabolites, and the down-facing arrow indicated the number of significantly decreased metabolites. (**B**) Sparse partial least squares discriminant analysis (sPLSDA) plots generated in Metaboanalyst. (**C**) Pie chart demonstrating the proportion of differentially altered metabolites by subtype. (**D**) Top 25 enriched pathways in the ileal mucosa of WT vs. SERT KO mice.

Functional changes in the gut microbiome generally manifest through alterations in metabolite abundances. Therefore, metabolomic analysis was performed as an unbiased approach to discovering novel changes in the intestinal mucosa during SERT deficiency that may explain the pleiotropic phenotype of SERT KO mice. The analysis revealed a unique metabolic signature in the ileal mucosa during the absence of SERT. Overall, there were 175 differentially altered metabolites (*p* < 0.05, q < 0.1), with 110 being decreased and 65 metabolites increased in the ileal mucosa of SERT KO mice compared to age and sex-matched WT mice (Figure 1A). Additionally, sparse partial least squares discriminant analysis (sPLSDA) revealed two distinct clusters when comparing the ileal mucosa metabolome of control mice to SERT KO mice (Figure 1B). Of the differentially altered metabolites, the largest proportion of altered metabolites was involved in lipid metabolism, with the next largest group involved in xenobiotic metabolism (Figure 1C). This assessment was followed up using enrichment analysis, which considers how many metabolites are altered within a given Kyoto Encyclopedia of Genes and Genomes (KEGG) pathway (Figure 1D). Of note, lipid pathways were amongst the most represented, including glycerophospholipid, linoleic acid, and fatty acid metabolism, to highlight a few.

### 2.2. Lack of SERT Results in Altered Mucosal Glycerols and Long Chain Monounsaturated Fatty Acids

**Figure 2:**
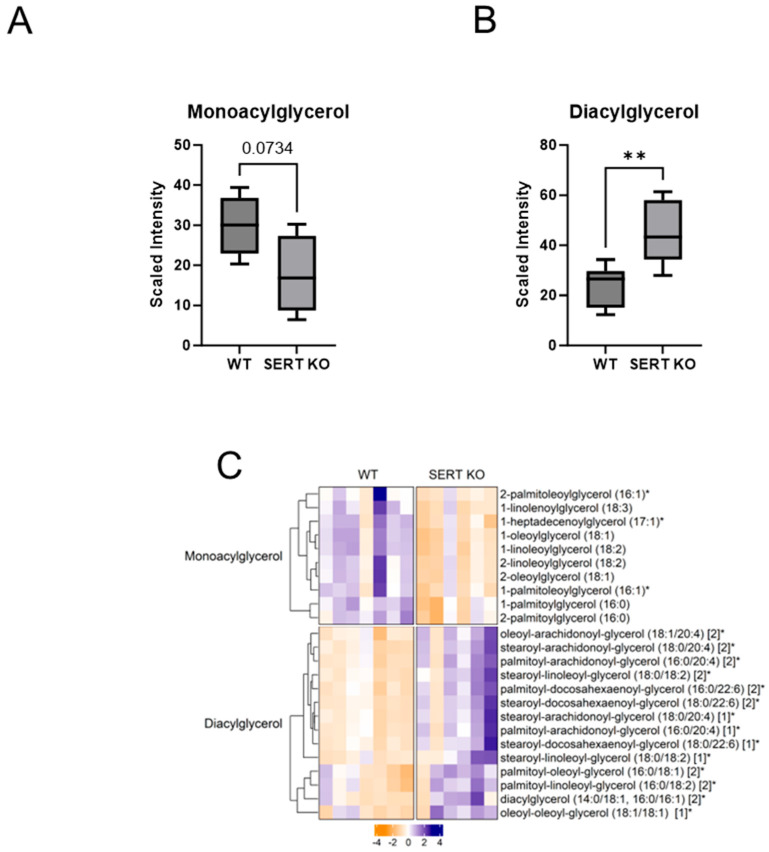
SERT KO mice demonstrate altered mucosal monoacylglycerol and diacylglycerol pools. (**A**) Boxplot of overall monoacylglycerol and (**B**) diacylglycerol abundances in ileal mucosa (Welch *t*-test; ** *p* < 0.01; WT n = 7; SERT KO n = 6) (**C**) Heatmap of differentially altered monoacylglycerol and diacylglycerol metabolites (*p* < 0.05, q < 0.1, Welch *t*-test; WT n = 7; SERT KO n = 6). * Indicates compounds with lack of reference standard acquisition but their identity is based upon orthogonal information. [1] and [2] indicates a compound that is a structural isomer of another compound in the Metabolon spectral library. In this instance, a diacylglycerol for which more than one stereospecific molecule exists.

**Figure 3:**
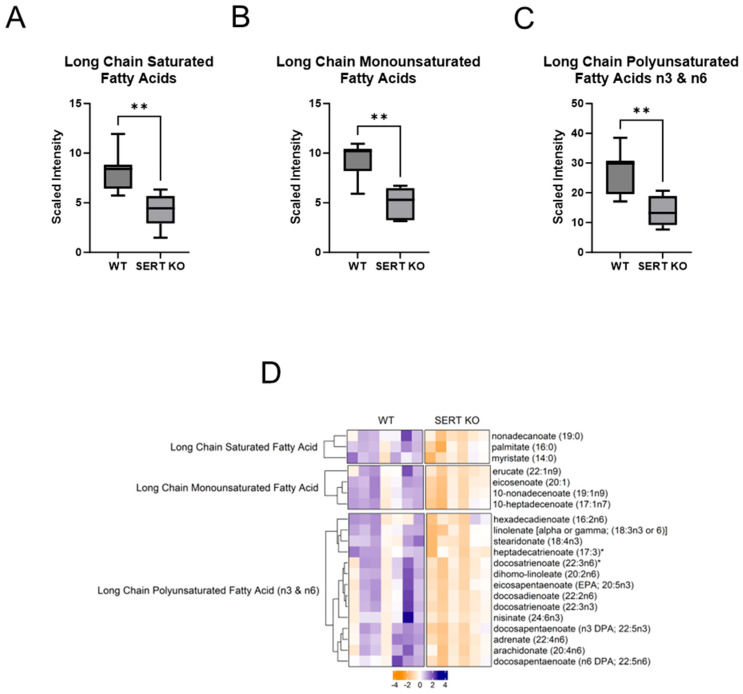
Long-chain fatty acids are depleted in ileal mucosa, which lacks SERT. (**A**) Boxplot of overall long-chain saturated fatty acids, (**B**) long-chain monounsaturated fatty acids, and (**C**) long-chain polyunsaturated fatty acids (n3 and n6) abundances in ileal mucosa (Welch *t*-test; ** *p* < 0.01; WT n = 7; SERT KO n = 6) (**D**) Heatmap of differentially altered long chain fatty acids (*p* < 0.05, q < 0.1, Welch *t*-test; WT n = 7; SERT KO n = 6). * Indicates compounds with lack of reference standard acquisition but their identity is based upon orthogonal information.

Fatty Acid and Phospholipid Metabolism Changes Due to SERT KO: Given that enrichment analysis and composition analysis identified lipids as a major chemical class altered in the ileal mucosa of mice lacking SERT, further analysis into lipid alterations was warranted. One source of substrates that are metabolized for energy is fatty acids. Fatty acids are cleaved from triacylglycerols (TAG, fats), diacylglycerols (DAG), and monoacylglycerols (MAG), yielding DAG, MAG, and glycerol, respectively, in addition to the cleaved fatty acid. Many saturated, monounsaturated, and polyunsaturated fatty acids, as well as monoacylglycerols, are altered in the ileum of SERT KO mice compared to control mice. When looking at the cumulative amounts of monoacylglycerol metabolites, there was a trend toward a decrease in SERT KO mice compared to WT (Figure 2A). Regarding diacylglycerols, there was a significant increase in mice lacking SERT (Figure 2B). Upon further analysis, those trends became evident, given that there were 10 significantly decreased monoacylglycerols and 14 increased diacylglycerols in SERT KO mice. Furthermore, given that various fatty acid pathways were identified in enrichment analysis, they were more closely examined (Figure 3). When grouping together different classes of fatty acids as a collective, there were significant decreases in long-chain saturated, monounsaturated, and polyunsaturated fatty acids in SERT-deficient mice (Figure 3A–C). In addition, the long-chain saturated fatty acids nonadecanoate, palmitate, and myristate significantly diminished the ileal mucosa of SERT KO mice (Figure 3D) along with others, including four monounsaturated and 14 polyunsaturated long-chain fatty acids that were also diminished.

Branched fatty acids (Figure S3A) showed a similar pattern with a significant decrease in collective abundance, with significant decreases in the levels of three metabolites in SERT KO mice ileal mucosa (Figure S3B). The decrease in several fatty acids observed in the SERT KO mice may be due to decreased production or absorption or increased utilization for anabolic needs, such as phospholipid production. Phospholipids are prevalent in lipid membranes and are necessary for cell growth and survival. The data show trends towards overall increases in phosphatidylcholines and phosphatidylethanolamines in SERT KO mice ileal mucosa (Figure S1A,B), with several individual phosphatidylcholine and phosphatidylethanolamine metabolites exhibiting increased levels in the ileal mucosa of SERT KO mice. (Figure S1C). On the other hand, there was a trend toward decreases in lysophospholipids (Figure S2A), with eight individual metabolites decreasing and one increasing in the SERT KO ileal mucosa (Figure S2B).

### 2.3. SERT KO Mice Exhibit Broad Changes in Metabolite Abundances

**Figure 4:**
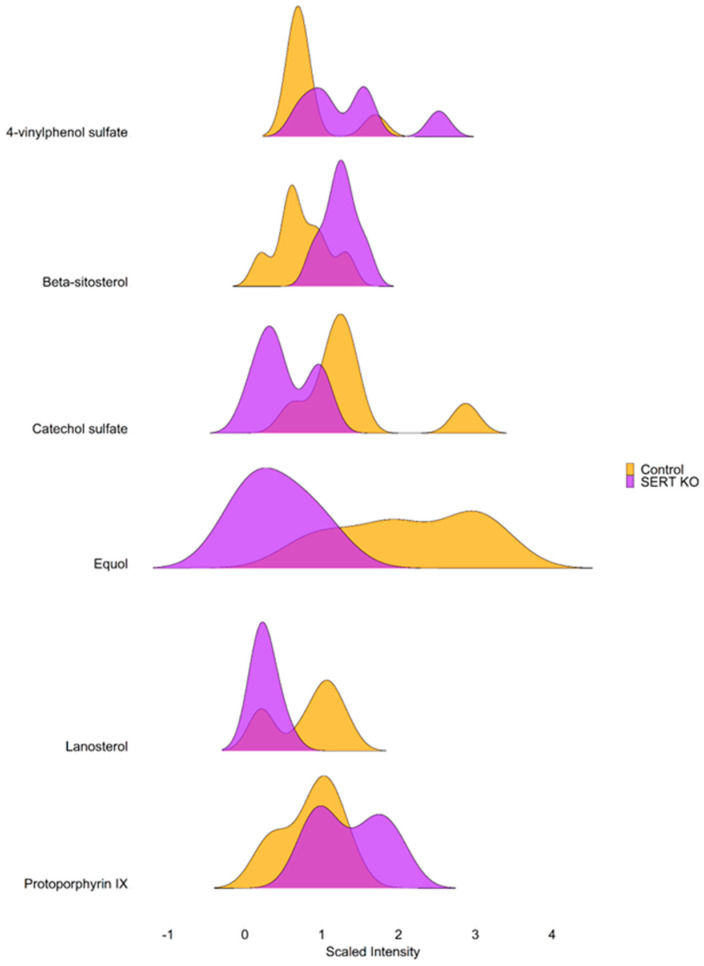
SERT leads to altered metabolite distributions. Ridgeline plot showing the distributions of differentially altered metabolites involved in dietary absorption and enterohepatic circulation. Ridgeline plots demonstrate the density of values along the *x*-axis in the form of an estimated probability density function. (*p* < 0.05, q < 0.1, Welch *t*-test; WT n = 7; SERT KO n = 6).

SERT KO mice demonstrated alterations in metabolites involved in enterohepatic circulation and dietary metabolites highlighted in Figure 4. For example, there was a significant increase in ileal levels of 4-vinylphenol sulfate in SERT KO mice, a compound that has been shown to have an association with the development of diabetic retinopathy [40]. Additionally, SERT KO mice showed diminished ileal mucosal levels of equol, a known bacterial metabolite produced in colonized hosts, that has been described as having beneficial estrogenic and antioxidant effects [41]. Furthermore, given the large quantity of altered metabolites, Met-Origin was utilized to group differentially altered metabolites (Figure S4). Interestingly, threonate and 4-hydroxybutyrate were increased in the ileal mucosa of mice lacking SERT. Other notable alterations included decreases in arabinose, equol, ferulate, ectoine, and biopterin. In line with enrichment analysis (Figure 1D), this analysis also revealed that sterol metabolites campesterol and beta-sitosterol were also increased in the ileal mucosa of mice lacking SERT. An increase in nucleotide metabolites, such as UDP-galactose, -glucose, and -glucuronate, in addition to adenosine, guanosine, uridine, and guanine, was also observed. Ultimately, this analysis highlights the vast influence of SERT on the ileal mucosal metabolite pool.

### 2.4. SERT KO Mice Exhibit Distinct Innate Lymphoid Cell Populations

**Figure 5:**
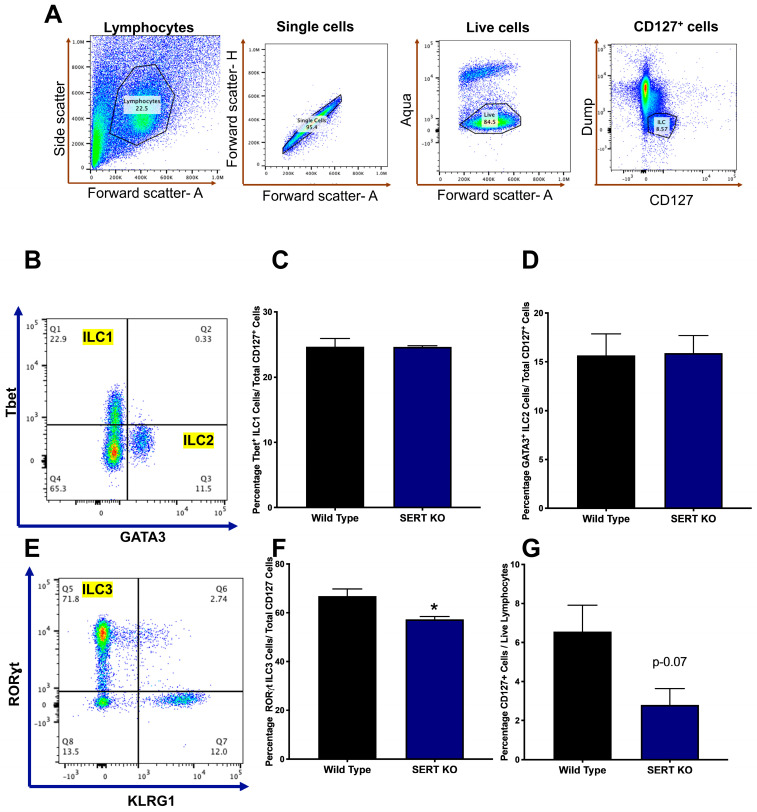
Lack of SERT leads to altered composition of intestinal innate lymphoid cells. Lamina propria lymphocytes were isolated from mice ileal tissues, and 1 million cells were stained for ILC transcription factors marking ILC1 (Tbet), ILC2 (GATA3), ILC3 (RORɣt) and Total CD127^+^ (**A**) Gating strategy in FACS analysis for CD127^+^ ILC. (**B**) FACS dot plots identifying ILC1 (Q1) and ILC2 (Q3) populations. (**C**) Representative bar diagram showing the percentage of ILC1 in total CD127^+^ Cell population. (**D**) Representative bar diagram showing the percentage of ILC2 in total CD127^+^ Cell population. (**E**) FACS dot plots identifying ILC3 (Q5) population. (**F**) Representative bar diagram showing the percentage of ILC3 in total CD127^+^ cell population. (**G**) Representative bar diagram showing the percentage of CD127^+^ cells in the total live lymphocytic cell population in the ileal lamina propria. Data represented as average ± SEM, n = 3, * *p* < 0.05 vs. WT, Welch *t*-test.

Prior investigations by our group have revealed that serotonin (5-HT) serves as an endogenous activator of the aryl hydrocarbon receptor (AhR). Thus, the absence of SERT impairs the activation of AhR [39]. The ileal mucosa of SERT KO mice demonstrated changes in tryptophan-derived AhR ligands xanthurenate, indoleacetylglycine, and kynurenate (Table 1). Given that AhR promotes ILC3 homeostasis, ILC populations were examined in the lamina propria of WT and SERT KO mice using fluorescence-activated cell sorting (FACS). In the CD127^+^ innate lymphocytic populations, there were no changes in the percentage of total ILC1 and ILC2 (Figure 5C,D). However, the ILC3 population, which is known to help maintain host-microbiota homeostasis, was significantly lower in SERT KO mice compared to WT littermates (Figure 5F). In addition, the total ILC population in the lamina propria also showed a trend towards a reduction in levels as compared to WT mice (Figure 5G). However, there were no changes in the adaptive CD4^+^T helper cell populations (Th1, Th2, Th17, and Treg) in the ileal lamina propria of wild-type and SERT KO mice (Figure S5).

## 3. Discussion

In this study, it was hypothesized that mice lacking serotonin transporter (SERT) exhibit intestinal mucosal alterations in lipid and microbial metabolites, providing additional clues into the roles of SERT in gastrointestinal physiology and disease. SERT-deficient mice have been shown to exhibit a high anxiety phenotype linked to increased extracellular availability of serotonin [42]. Furthermore, SERT KO mice develop a metabolic syndrome-like phenotype that includes increased leptin, glucose intolerance, insulin resistance, obesity, and hepatic steatosis as they age [23]. The SERT KO model has also been shown to have an enhanced susceptibility to intestinal inflammation [26,27]. It is also notable that SERT expression has been found to be decreased in inflammatory bowel disease, during a major depressive episode, and in type-2 diabetes [25,43,44]. This study explored the ileal mucosal metabolome of SERT knockout (KO) mice, revealing a distinctive metabolomic signature characterized predominantly by alterations in lipid metabolites. To compare classes of metabolites, the levels of all metabolites belonging to a particular class were summed. SERT KO mice broadly manifested decreased levels of monoacylglycerols and increased levels of diacylglycerols when compared to age and sex-matched wild-type (WT) mice. Additionally, SERT KO mice had several decreases in multiple classes of long-chain fatty acids, including saturated, monounsaturated, and n3 and n6 polyunsaturated. Furthermore, SERT deficiency led to altered abundances of other notable metabolites in the ileal mucosa, including several dietary metabolites and metabolites in enterohepatic circulation. Moreover, further investigation revealed a diminished proportion of type 3 innate lymphoid cells (ILC3) in the lamina propria of SERT KO mice when compared to WT.

### 3.1. Facts and Perspectives

Gut microbial dysbiosis is a hallmark of the global SERT deficiency [28,45]. More specifically, these changes have been associated with gene expression changes suggestive of altered host lipid metabolism [28]. This study further explored those previous observations by taking an unbiased approach through the measurement of a multitude of ileal mucosal metabolites, revealing that the largest subset of altered metabolites were lipids. Notable changes included decreased levels of mucosal monoacylglycerols and increased levels of diacylglycerols in SERT KO mice. It is known that triacylglycerols are broken down by lipases in the lumen of the small intestine to produce free fatty acids and absorbable monoacylglycerols [46]. Previous work has shown that genes involved in the biosynthesis of unsaturated FAs are upregulated in SERT KO mice, including HMGCS2 [28]. The increased presence of diacylglycerols and concurrent diminished levels of monoacylglycerols suggests that there is impaired triacylglycerol breakdown into monoacylglycerols in fatty acids, alluding to the possibility that there is impaired enteric lipid breakdown and subsequent absorption in the absence of SERT. Alternatively, these findings can also suggest alterations in the synthesis of triacylglycerides from monoglycerides. Interestingly, previous studies have shown that SERT KO mice have increased absorption of simple carbohydrates and medium-chain fatty acids, which is attributed to potentiated 5-HT signaling [47]. Although there were no differences in measured medium chain fatty acid mucosal levels between WT and SERT KO mice in this study, both observations suggest alterations in intestinal lipid absorption in the absence of SERT. Moreover, diacylglycerols are also known to serve as signaling molecules as secondary messengers, playing key roles in both innate and adaptive immunity [48]. Notably, increased intracellular diacylglycerol levels are also known to contribute to insulin resistance, a known characteristic feature of SERT KO mice [22,23,49]. Furthermore, increased serum diacylglycerol levels have been implicated in human metabolic syndrome, type 2 diabetes, and prediabetes [49,50,51]. It is also worth noting that, to our knowledge, no studies were found in which small intestinal metabolomics have been performed in human subjects with metabolic syndrome and related disorders, making it challenging to put these findings in the context of previous lipidomic studies. Additionally, SERT KO mice demonstrated increased ileal mucosa levels of 4-hydroxybutyrate. While not a direct link, the observation that 3-hydroxybutyrate dehydrogenase type 2—which initiates the breakdown of 3-hydroxybutyrate is increased in SERT KO ileal mucosa—suggests a physiological deviation in how ketones are utilized as an energy source in the absence of SERT [39,52]. This offers a new clue into the interplay between serotonergic signaling and insulin resistance, suggesting that it may be due at least in part to increased abundances of diacylglycerols.

Further analysis revealed several changes in compounds related to diet and enterohepatic communication in SERT KO mice. For instance, beta-sitosterol, a phytosterol that is known to improve induced dextran sulfate sodium-induced colitis [53] and inhibit obesity-induced insulin resistance, [54] is increased in the mucosa of SERT KO mice. It is known that SERT KO mice are prone to developing obesity and insulin resistance [23]. The observation that these phytosterols are increased in the ileal mucosa indicates perturbed intestinal handling of these compounds in the case of SERT deficiency, and the underlying mechanisms will be investigated in future studies. The isoflavone equol had a significantly decrease in the ileal mucosa in SERT KO mice compared to WT mice. Previous studies have shown the beneficial effects of equol on insulin resistance, but more recently, it has been shown that there is a lower incidence of diabetes and dyslipidemia in human females whose gut microbiota produces equol [55,56]. Taken together, along with the fact that equol production is dependent on incompletely characterized gut microbiota [41], this suggests that known gut microbial dysbiosis in SERT KO contributes to insulin resistance partially through a lack of equol production. Lanosterol, a cholesterol precursor, was also decreased in the abundance of SERT KO ileal mucosa compared to WT. Interestingly, there is some evidence that lanosterol serves as a functional insulin analog by promoting glucose uptake in 3T3-L1 adipocytes [57]. While SERT-deficient mice are known to develop insulin resistance and metabolic syndrome, they are also known to have increased susceptibility to intestinal inflammation [26,27]. Ectoine and arabinose, microbial metabolites that have been shown to attenuate intestinal inflammation [58,59,60], are also decreased in the ileal mucosa of SERT-deficient mice, offering additional insight into how serotonergic dysregulation can increase susceptibility to intestinal inflammation. Collectively, these findings demonstrate a complex interplay between intestinal serotonergic signaling and mucosal metabolites that play key roles in insulin homeostasis and intestinal inflammation while offering novel avenues for future investigations examining how intestinal SERT affects host physiology related to lipid and nutrient absorption.

One such avenue this present study explored was how SERT deficiency alters intestinal immune populations, which are known to affect host physiology in healthy and diseased states. While this work did not find any differences in the abundance of Th1, Th2, Th17, or Treg cells in the ileal lamina propria of SERT-deficient mice, this analysis uncovered diminished levels of group 3 innate lymphoid cells (ILC3) in the absence of SERT. Given previous literature, it is likely that diminished levels of ILC3 cells in the absence of SERT may contribute to the pleiotropic phenotype that is generated during SERT KO. For instance, hepatic ILC3s have been shown to protect against steatohepatitis in mice fed with a high-fat diet [61]. Furthermore, ILC3 cells have also been shown to play important roles in mucosal immunity, such as protecting the host during *Citrobacter rodentium* infection [62]. ILC3 cells are known to respond to several physiologic signals, such as growth factors, cytokines, commensal microbes, and metabolites, to help maintain intestinal epithelial barrier function, aid in intestinal absorption, and modulate adaptive immunity [63,64]. Thus, it is not surprising that given how SERT KO mice are predisposed to the development of metabolic syndrome and increased susceptibility to intestinal inflammation, they would exhibit diminished levels of ILC3 cells. While there are diminished levels of ILC3 cells, it is important to note that this observation is not sufficient evidence to suggest that there is diminished ILC3 signaling or function, but it warrants future investigations into how ILC3 functions are affected by serotonergic signaling. Previous work by our group demonstrated decreased expression of AhR target genes in SERT KO ileal mucosa, likely due to diminished intracellular serotonin and diminished AhR activation via an indirect mechanism [39]. This work further showed that during SERT deficiency in vivo, potent AhR ligands (B-naphtha flavone and indole-3-carbinol) were unable to raise CYP1A1 levels—a canonical AhR target gene—demonstrating that serotonin-mediated AhR activation is SERT dependent. Interestingly, SERT KO mice ileal mucosa demonstrated decreases in several AhR-activating tryptophan metabolites, including xanthurenate, indoleacetylglycine, and kynurenate. While not significantly different, SERT KO mice demonstrated a ~3-fold increase in the ileal mucosa levels of 5-HT and a significant depletion in relative abundance of plasma 5-HT levels (SERT KO:WT 0.11) as expected. Given that AhR signaling is known to maintain ILC3 populations in the intestines [65], the findings offer one possibility into why ILC3 levels were diminished in SERT KO intestines. Furthermore, given that ILC3 cells respond to various physiological signals such as growth factors, cytokines, and metabolites, it is plausible that altered levels of AhR activating tryptophan metabolites could directly or indirectly affect these signaling cascades, thereby influencing ILC3 function. Future studies can focus on investigating AhR target gene cross-talk in epithelial and immune cells, specifically in ILC3 cells derived from SERT KO mice. Such experiments could shed light on the underlying mechanisms of ileal ILC3 diminishment in SERT-deficient mice and further elucidate the interplay between serotonin signaling, metabolic pathways, and intestinal immune regulation.

### 3.2. Strengths of the Study

This study utilized an in vivo mouse model to study SERT signaling and explored metabolomics in an often-neglected tissue compartment, the ileal mucosa. Moreover, this work employed an unbiased discovery approach to identifying and exploring novel mucosal metabolomic changes in the absence of SERT. This led to the identification of several new areas of investigation with implications for metabolic syndromes and intestinal inflammation, such as how potentiation of 5-HT signaling alters lipase activity, function, and abundance. This work also provides a foundation for further exploring how innate lymphoid cells, specifically in the small intestine, interact with serotonergic signaling with applications for metabolic, infectious, and inflammatory gut disorders. Limitations of this study include that it only utilized male mice and should be expanded in the future to include female mice at various stages of their estrous cycle. Future studies can focus on the utilization of targeted metabolomics with further stratification to include metabolite measurements in intestinal epithelial cells and in the lamina propria to better characterize the crosstalk between luminal microbes, intestinal epithelial cells, and intestinal immune cells. Additionally, the bioinformatic tool Met-Origin utilized previously published literature to identify compounds as microbial in origin. These classifications require individual validation but were used simply for organization and grouping purposes to highlight notable differentially altered metabolites. Finally, future mechanistic studies would also benefit from the use of inducible, intestinal-specific gene manipulations as opposed to the global SERT KO model that was utilized.

## 4. Materials and Methods

### 4.1. Animals

All animal studies were performed in accordance with institutional guidelines and regulations and as approved by the Animal Care Committees of the University of Illinois at Chicago and the Jesse Brown Veterans Affairs Medical Center. Wild-type C57/BL6 mice were purchased from Jackson Laboratory (Bar Harbour, ME, USA). SERT KO heterozygous mice were purchased from Jackson Laboratory (B6.129(Cg)-Slc6a4tm1Kpl/^J(+/+)^; strain # 008355). Autoclaved polypropylene cages with corncob bedding were used to house the mice. The mice were given free access to food and water (Teklad Irradiated LM-485 Mouse/Rat Diet 7912, Envigo (Indianapolis, IN, USA)) under a 12-h light/dark cycle. Male mice aged (7–9 weeks) were utilized.

### 4.2. Metabolomic Analysis

Ileal mucosal scrapings were processed and analyzed by Metabolon Inc. (Durham, NC, USA) as previously described [66,67,68]. Further detail is provided below.

#### 4.2.1. Sample Preparation

After euthanasia, the intestines were immediately resected, and the ileum was isolated. Mucosa was scraped, flash-frozen in liquid nitrogen, and stored at −80 °C. Samples were prepared with Hamilton Company’s automated MicroLab STAR^®^ system. Recovery standards were added prior to extraction for quality control. Proteins were precipitated with methanol, followed by centrifugation to dissociate small molecules bound to protein or trapped in the precipitated protein matrix (Glen Mills GenoGrinder 2000). The extract was divided into five fractions (one for backup) for analysis using (RP)/UPLC-MS/MS methods with positive ion mode electrospray ionization (ESI), RP/UPLC-MS/MS with negative ion mode ESI, and HILIC/UPLC-MS/MS with negative ion mode ESI. Organic solvents were removed using TurboVap^®^ (Zymark), and samples were dried under a nitrogen gas [66,67,68].

#### 4.2.2. Ultrahigh Performance Liquid Chromatography-Tandem Mass Spectroscopy (UPLC-MS/MS)

For Ultrahigh Performance Liquid Chromatography-Tandem Mass Spectroscopy (UPLC-MS/MS), Waters ACQUITY UPLC and Thermo Scientific Q-Exactive mass spectrometer were used. Sample extracts were reconstituted in appropriate solvents with standards for four analysis methods. One aliquot was analyzed under acidic positive ion conditions, which were further chromatographically adjusted for more hydrophilic substances. In this case, the extract was eluted via gradient using water and methanol containing 0.05% perfluoropentanoic acid (PFPA) and 0.1% formic acid (FA) in a C18 column with the following specifications: Waters UPLCA BEH C18-21.1 × 100 mm, 1.7 m.

Another aliquot was utilized for examination under acidic positive ion conditions that were chromatographically adjusted for more hydrophobic substances in the same column described above, with the same solvent compositions, and operated at a higher total organic content. Another C18 column was utilized for examination under basic negative ion conditions. The basic extracts were eluted via gradient with methanol and water containing 6.5 mM ammonium bicarbonate at pH 8 from an HILIC column with the following specifications: Waters UPLC BEH Amide 2.1 × 150 mm, 1.7 m. The fourth aliquot was utilized for negative ionization analysis and eluted via gradient with water and acetonitrile with 10 mM ammonium formate, pH 10.8. The raw data files were archived and extracted as described below [66,67,68].

#### 4.2.3. Compound Identification and Metabolite Quantification

Compound identification and metabolite quantification were conducted using Metabolon’s (Durham, NC, USA) equipment and software. Raw data were processed, peaks were identified by comparing them to a library of purified standards, and compounds were identified based on retention index, accurate mass match, and MS/MS data. Peak measurements were normalized to total protein [66,67,68].

### 4.3. Lamina Propria Lymphocyte Isolation

The whole ileum was flushed with 1X PBS to remove feces. The tissue was stripped of all fat and Peyer’s patches. Next, the ileum was opened longitudinally and cut into small pieces, then washed in 5 mM EDTA containing PBS with 1 mM DTT at 37 °C for 20 min in an orbital shaker at 225 rpm to remove mucus. Then, the solution was passed through a 100 μm strainer, and the solution was replaced without DTT and again was shaken for 10 more min. Then, the solution was strained, and tissue digestion was conducted with 1 mL digestion buffer prepared in complete media containing Dispase (0.1 units/mL), Collagenase-D (1 mg/mL), and DNAse (0.1 mg/mL). The tissues were minced using a scissor and then transferred to a conical tube with 7 mL digestion buffer and shaken at 37 °C for 20 min in an orbital shaker at 225 rpm. Finally, the tissues were strained quickly through a 100 μm cell strainer, and the eluent was centrifuged at 2200 rpm for 7 min to collect the cell pellet. Next, the cell pellet was dissolved in 40% percoll, overlayed with 80% percoll, and centrifuged at 2200 rpm with no breaks for 22 min. The intermediate phase enriched with lymphocytes was collected into complete media and counted to perform cell staining.

### 4.4. Flow Cytometry

Cell staining was performed in 1 million cells per panel for CD4^+^ T helper cells and CD127^+^ innate lymphoid cell (ILC) populations. Cell surface and transcription factor staining were conducted using bioscience reagents (San Diego, CA, USA) according to the manufacturer’s protocol. Antibodies used from Biolegend (San Diego, CA, USA) were incubated per the ebio protocol in specified buffers at a 1:200 ratio for cell surface markers, including CD4, CD127, TCRβ, CD44, CD8, MHCII, CD3e, CD11b, CD14, CD19, B220, TCRγδ, NK1.1, and KLRG1. Antibodies for transcription factors (Tbet, FOXP3, RORγt, GATA3) were incubated at a ratio of 1:100. The stained cells were finally strained with a 40 μm cell strainer and were then run in Attune NxT flow cytometer (Thermo Fisher Scientific, Waltham, MA, USA). The samples were analyzed using FlowJo (v10.10) software (Franklin Lakes, NJ, USA) with specific gating strategies to identify various ILC and T cell populations.

### 4.5. Bioinformatic Analysis

Bioinformatic analysis was performed as previously described [67]. Briefly, statistically significant differential metabolites were displayed utilizing heatmaps that were generated with ComplexHeatMap in RStudio (version 2023.12.1 + 402) [69,70]. Met-origin was utilized to identify microbial-related metabolites [71]. It is important to note that MetOrigin functions through the integration of various metabolite databases such as the Kyoto Encyclopedia of Genes and Genomes and the human metabolome database (HMDB) that include source information including (mammals, microbiota, cometabolism, food, drug, and environment) and thus these classifications are based upon previous reports which require independent verification for rigor. Additionally, the MetOrigin computational pipeline is not freely available, which should also be taken into account by readers. This is mentioned in the limitations section of the discussion section.

Further, Metaboanalyst was utilized to perform enrichment and sparse partial least squares discriminant (sPLSDA) analysis. Metabolites were imported into Metaboanalyst with both the Kyoto Encyclopedia of Genes and Genomes (KEGG) and Human Metabolome Database (HMBD) IDs for sPLSDA analysis. Additionally, pathway enrichment analysis was performed based upon differentially altered metabolites in order to determine which metabolic pathways were most altered based upon changes in individual metabolites pertaining to specific defined metabolic pathways [72].

## 5. Conclusions

In summary, this study provides a mucosal metabolomic landscape that occurs during the absence of SERT and offers novel insights into how a decrease in SERT function may contribute to insulin resistance and increased intestinal inflammation. This study identified impaired triacylglycerol metabolism in the absence of SERT, diminished levels of anti-inflammatory microbial-derived metabolites, and diminished levels of intestinal ILC3 cells in the ileal mucosa. Finally, this work paves the way for future studies investigating the complexity between mucosal immunity, mucosal metabolites, gut microbiota, and serotonergic signaling in metabolic, infectious, and inflammatory gastrointestinal diseases.

## Figures and Tables

**Table 1:** Relevant fold changes in tryptophan-derived potential aryl hydrocarbon receptor (AhR) activators. List of selected metabolites, including tryptophan, serotonin, and tryptophan-derived metabolites in the ileal mucosa of SERT KO compared to WT mice. (Welch *t*-test; * *p* < 0.05, ** *p* < 0.01; WT n = 7; SERT KO n = 6).

Name	Fold Change Relative Abundance	*p*-Value	q-Value
Tryptophan	0.78	0.1489	0.1998
Serotonin	3.15	0.1147	0.1833
Indoleacetate	0.69	0.0987	0.1671
Xanthurenate	0.53 *	0.0406	0.1236
Indoleacetylglycine	0.4 **	0.0023	0.09
Kynurenate	0.6 **	0.0088	0.09

## Data Availability

Additional data is publicly available as part of supplemental data.

## Supplementary Materials

The supporting information can be downloaded at: https://www.mdpi.com/article/10.3390/ijms25084459/s1.
