# Supplementary material for: Serotonin Transporter Deficiency Induces Metabolic Alterations in the Ileal Mucosa
Source: Int J Mol Sci. 2024 Apr 18;25(8):4459. doi: 10.3390/ijms25084459 (PMC11049861; doi:10.3390/ijms25084459)
Supplement: Supplementary file 1 [file ijms-25-04459-s001.zip › ijms-2933895-supplementary.pdf]

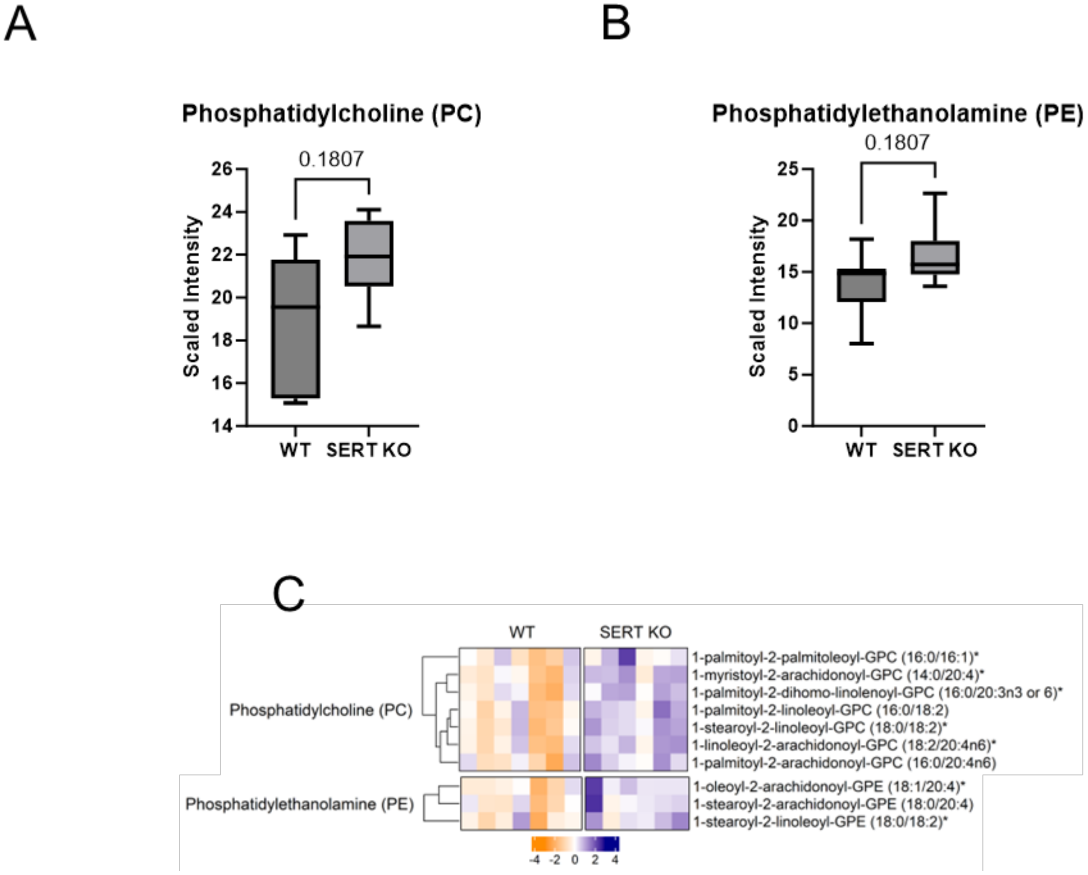

Supplemental Figure S1: **SERT KO mice have altered phosphatidylcholine (PC) and phosphatidylethanolamine (PE) metabolites. A.** Boxplot of overall phosphatidylcholine (PC) and **B.** phosphatidylethanolamine (PE) abundances in ileal mucosa (Welch *t*-test; WT $n = 7$; SERT KO $n = 6$) **C.** Heatmap of differentially altered PC and PE metabolites ($p < 0.05$, $q < 0.1$, Welch *t*-test; WT $n = 7$; SERT KO $n = 6$).

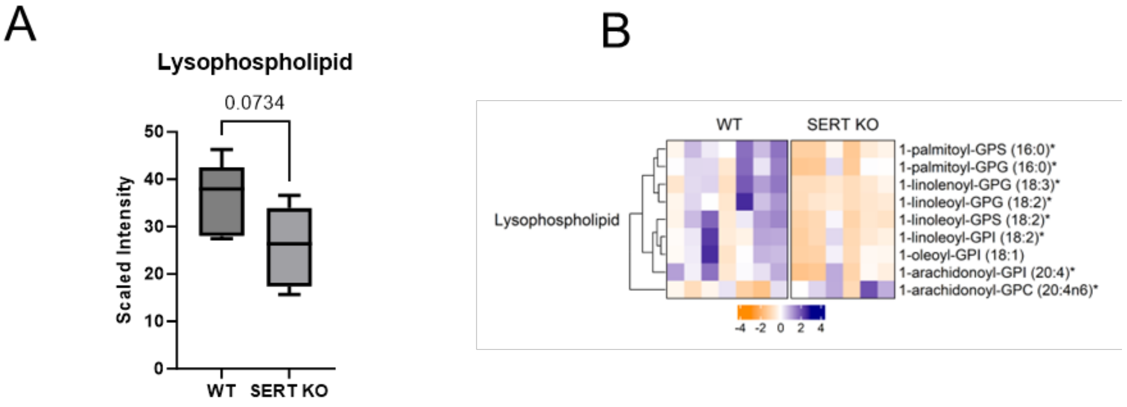

Supplemental Figure S2: **Lysophospholipid metabolites are diminished in SERT KO ileal mucosa. A.** Boxplot of overall lysophospholipid abundances in ileal mucosa (Welch *t*-test; WT $n = 7$; SERT KO $n = 6$) **B.** Heatmap of differentially altered lysophospholipid metabolites ($p < 0.05$, $q < 0.1$, Welch *t*-test; WT $n = 7$; SERT KO $n = 6$).

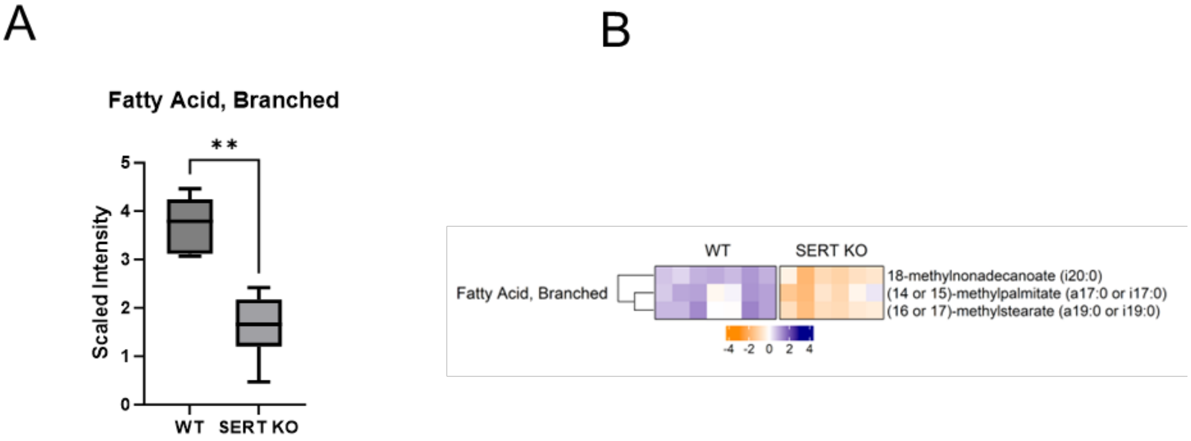

Supplemental Figure S3: **Ileal mucosa lacking SERT demonstrates diminished levels of branched fatty acids. A.** Boxplot of overall branched fatty acid abundances in ileal mucosa (Welch *t*-test; WT $n = 7$; SERT KO $n = 6$) **B.** Heatmap of differentially altered branched fatty acid metabolites ($p < 0.05$, $q < 0.1$, Welch *t*-test; WT $n = 7$; SERT KO $n = 6$). ** $p < 0.01$

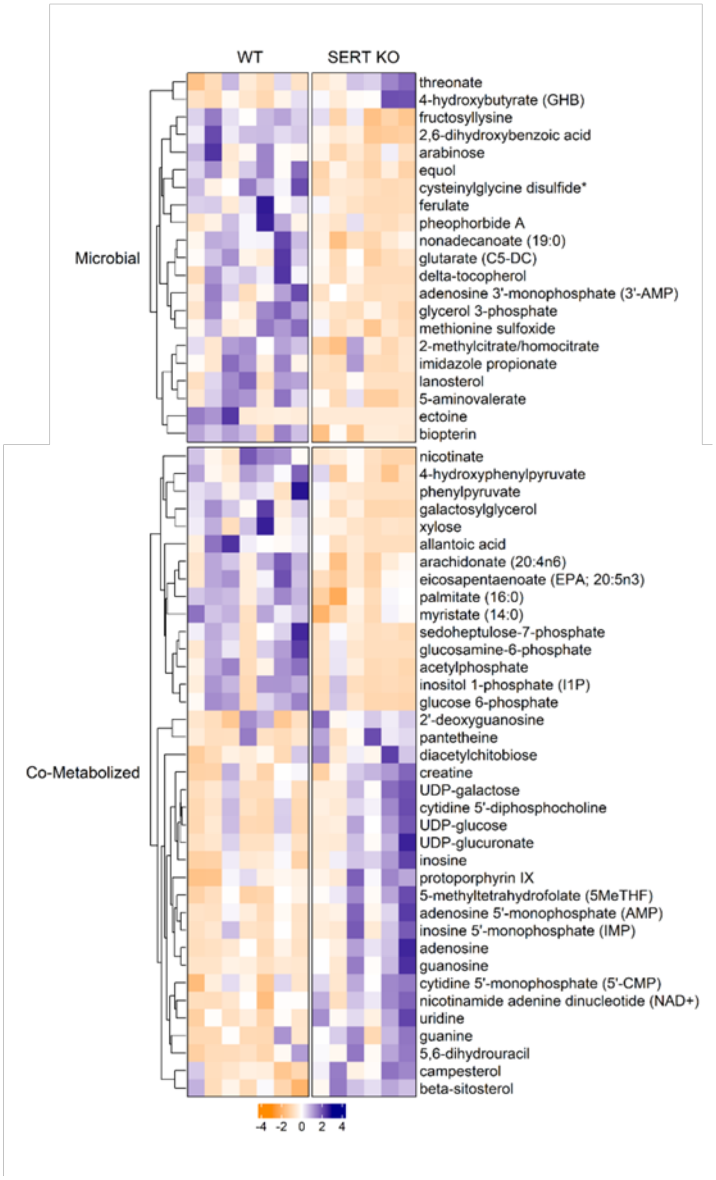

Supplemental Figure S4: **Heatmap of microbial-related and co-metabolized differentially altered metabolites between WT and SERT KO mice in the ileal mucosa**. Metabolites were classified based upon MetOrigin classification. ($p < 0.05$, $q < 0.1$, Welch t-test; WT $n = 7$; SERT KO $n = 6$).

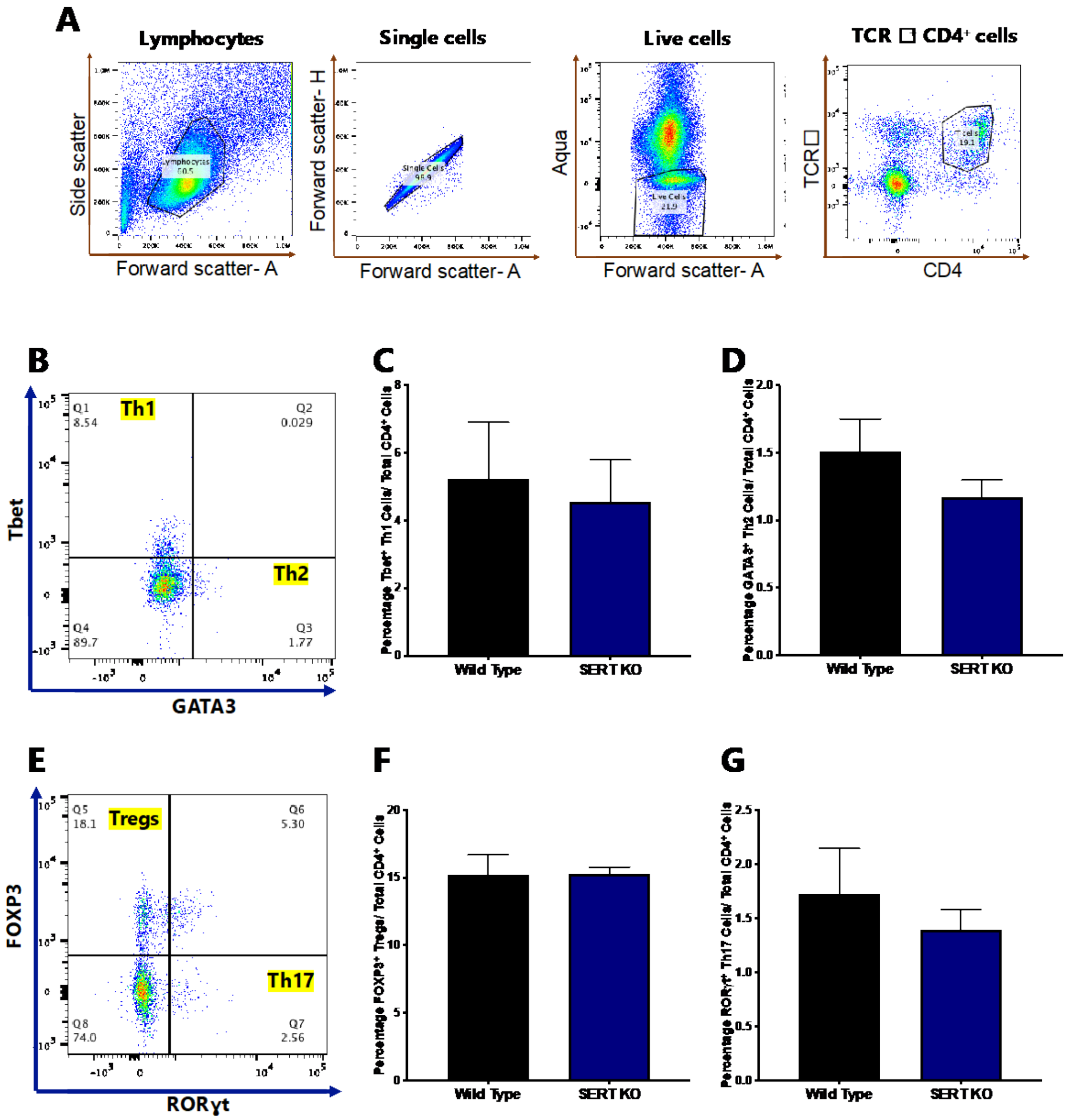

Supplemental Figure S5: **Various CD4+ T cell populations in Ileal lamina propria of wild type and SERT KO mice.** Lamina propria lymphocytes were isolated from mice ileal tissues and 1 million cells were stained for Th cell transcription factors marking Th1 (Tbet), Th2 (GATA3), Th17 (RORγt) and T regulatory cells (FOXP3) **A.** Gating strategy in FACS analysis for $CD4^+$ T cells. **B.** FACS dot plots identifying Th1 (Q1) and Th2 (Q3) populations. **C**. Representative bar diagram showing percentage of Th1 cells in total $CD4^+$ Cell population. **D.** Representative bar diagram showing percentage of Th2 cells in total $CD4^+$ Cell population. **E.** FACS dot plots identifying Tregs (Q5) and Th17 (Q7) populations. **F.** Representative bar diagram showing percentage of Treg cells in total $CD4^+$ Cell population. **G.** Representative bar diagram showing percentage of Th17 cells in total CD4+ cell population in the ileum. Data represented as average ± SEM, n=3.
